# P29 A nudge to safe dosing and sound sleeping: using visual cues to improve safety and sleep quality of patients prescribed intermittent IV vancomycin in the acute hospital setting

**DOI:** 10.1093/jacamr/dlag102.035

**Published:** 2026-06-26

**Authors:** Rachael Rodger, Hiera Qadri, Jonathan Yates

**Affiliations:** Royal Alexandra Hospital, Paisley, NHS Greater Glasgow & Clyde, UK; Royal Alexandra Hospital, Paisley, NHS Greater Glasgow & Clyde, UK; Royal Alexandra Hospital, Paisley, NHS Greater Glasgow & Clyde, UK

## Abstract

**Background:**

In NHS Greater Glasgow and Clyde (NHSGGC) reducing unnecessary night-time infusions of IV vancomycin has been an ongoing focus of quality improvement (QI).^1,2,4^ Prolonged overnight infusions can significantly impact sleep quality, an important factor in fighting infection, and compromise patient safety due to reduced night-time staffing and resources.^3^ Pragmatic guidelines introduced to promote a ‘Good Night’s Sleep’ for patients and manage missed and delayed IV vancomycin doses, significantly increased on time doses from 58% to 77%, however, nearly a quarter (23%) of doses were still given between midnight and 6 am.^4^ Nudge theory is a subtle approach to changing behaviour by guiding rather than mandating better decisions.^5^ Visual cues such as colour-coding and symbols can be highly effective as they are easier to understand than textual information, especially in fast-paced environments like busy wards. Visual cues work best when placed in close proximity to the decision making. NHSGGC intermittent IV vancomycin prescribing administering and monitoring (PAM) charts were recently updated with visual cues (colour-coded blue prescribing boxes and moon symbols) to direct prescribing and administration times away from the middle of the night.^1^

**Objectives:**

To determine if a QI nudge approach, adding visual cues directly to the NHSGGC intermittent IV vancomycin PAM chart, can reduce the percentage of doses prescribed between midnight and 6am at the Royal Alexandra Hospital (RAH) to less than 10% by January 2026.

**Methods:**

A piloted and standardized Microsoft Forms data collection tool and electronic prescribing (HEPMA) reports, were used to collect prospective baseline data (Jan 2023-Dec 2023) for RAH adult inpatients prescribed intermittent IV vancomycin. The process was repeated ‘post change’ (Jan 2025-Dec 2025) 6 months after introduction of the updated PAM charts. The proportion of intermittent IV doses prescribed between midnight and 6am was collated pre and post change and displayed on run charts to measure improvement.

**Results:**

In total 148 patients (55% male) with a mean age of 67 years from medical (38%), surgical (55%) and elderly care (7%) wards, prescribed intermittent IV vancomycin, were prospectively reviewed. The majority of patients had IV vancomycin treatment durations between 2-13 days (78%), with a small proportion less than 48 h (16%) or more than 14 days (6%). Common indications were bone/joint infections, skin/soft tissue infections and bacteraemia. At baseline (Jan 2023-Dec 2023) the median proportion of intermittent IV vancomycin doses (*n*=764) prescribed between midnight and 6am was 18% (Figure 1). Post change (Jan 2025–Dec 2025) the median proportion of maintenance doses (*n*=354) prescribed within this time window was significantly reduced to a median of 7% (Figure 1), meeting the QI target set.

**Conclusions:**

Using a QI nudge approach to introduce visual cues directly at the time of prescribing resulted in a sustained change in prescribing behaviour and significantly reduced the median proportion of intermittent IV vancomycin doses prescribed in the middle of the night from 18% to 7%. This is important in promoting person-centred care^6^ and improving sleep quality and safety in patients receiving intermittent IV vancomycin to treat infections in hospital.

**Figure 1.**
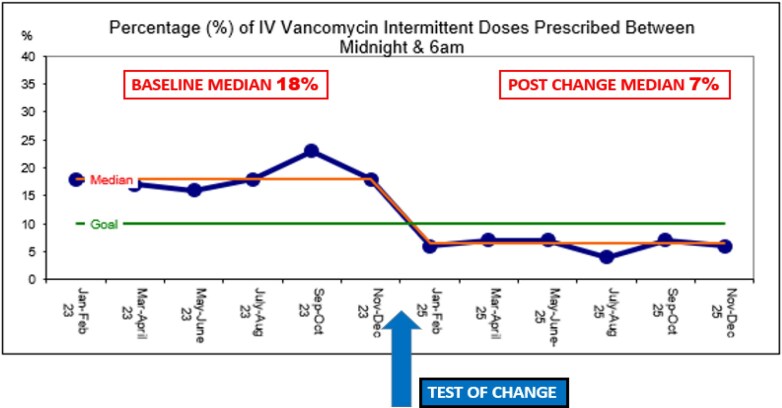
The percentage (%) of IV vancomycin doses prescribed between midnight and 6am in the Royal Alexandra Hospital pre (*n*=764) and post (*n*=354) introduction of updated PAM charts (Test of Change) incorporating visual cues to direct choice of selected prescribing times.
